# Porous Cellulose Thin Films as Sustainable and Effective Antimicrobial Surface Coatings

**DOI:** 10.1021/acsami.2c23251

**Published:** 2023-03-29

**Authors:** Shaojun Qi, Ioannis Kiratzis, Pavan Adoni, Aekkachai Tuekprakhon, Harriet James Hill, Zania Stamataki, Aneesa Nabi, David Waugh, Javier Rodriguez Rodriguez, Stuart Matthew Clarke, Peter J. Fryer, Zhenyu J. Zhang

**Affiliations:** † School of Chemical Engineering, University of Birmingham, Birmingham B15 2TT, U.K.; ‡ Institute of Immunology and Immunotherapy, University of Birmingham, Birmingham B15 2TT, U.K.; § School of Mechanical, Aerospace and Automotive Engineering, Coventry University, Coventry CV1 2JH, U.K.; ∥ Yusuf Hamied Department of Chemistry, Cambridge University, Cambridge CB2 1EW, U.K.

**Keywords:** cellulose, film, antimicrobial, evaporation, SARS-CoV-2, robustness

## Abstract

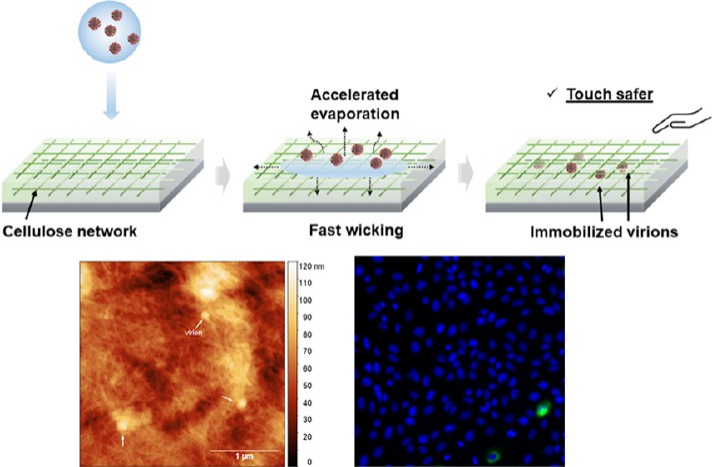

In the present work, we developed an effective antimicrobial surface film based on sustainable microfibrillated cellulose. The resulting porous cellulose thin film is barely noticeable to human eyes due to its submicrometer thickness, of which the surface coverage, porosity, and microstructure can be modulated by the formulations and the coating process. Using goniometers and a quartz crystal microbalance, we observed a threefold reduction in water contact angles and accelerated water evaporation kinetics on the cellulose film (more than 50% faster than that on a flat glass surface). The porous cellulose film exhibits a rapid inactivation effect against SARS-CoV-2 in 5 min, following deposition of virus-loaded droplets, and an exceptional ability to reduce contact transfer of liquid, e.g., respiratory droplets, to surfaces such as an artificial skin by 90% less than that from a planar glass substrate. It also shows excellent antimicrobial performance in inhibiting the growth of both Gram-negative and Gram-positive bacteria (*Escherichia coli* and *Staphylococcus epidermidis*) due to the intrinsic porosity and hydrophilicity. Additionally, the cellulose film shows nearly 100% resistance to scraping in dry conditions due to its strong affinity to the supporting substrate but with good removability once wetted with water, suggesting its practical suitability for daily use. Importantly, the coating can be formed on solid substrates readily by spraying, which requires solely a simple formulation of a plant-based cellulose material with no chemical additives, rendering it a scalable, affordable, and green solution as antimicrobial surface coating. Implementing such cellulose films could thus play a significant role in controlling future pan- and epidemics, particularly during the initial phase when suitable medical intervention needs to be developed and deployed.

## Introduction

A range of infectious pathogens, including respiratory viruses and disease-causing bacteria, can spread as surface fomites.^1^ The transmission of SARS-CoV-2, for example, might take place when a human makes contact with a surface that is contaminated by infectious respiratory secretions or droplet nuclei,^2^ despite that air transmission has been recognized as the primary route for SARS-CoV-2. In a recent study by Public Health England, it was reported that viable virus persisted for long periods of time on hydrophobic surfaces, e.g., surgical masks and stainless steel up to 7 days.^1^ In a contact transfer context, studies showed that a contact of 5 s is adequate to transfer 31.6% of an influenza virion load to the counter surface^3^ or approximately 15% of SARS-CoV-2 through a light touch by a finger.^4^

Chemical disinfectants, such as conventional bleach with appropriate dilution,^5^ ethanol, and treatment with dried hydrogen peroxide,^6^ are commonly used to ensure surface hygiene with an effect in minutes,^6−8^ many of which contain potentially harmful substances such as chlorine bleach, phenolics, and quaternary ammonium compounds when excessive quantity is used.^9,10^ For example, chlorine disinfectants irritate the mucous membranes of the respiratory and digestive systems^11^ and can cause acute toxicity or even death to both terrestrial and aquatic wildlife.^12^ The prevailing practice of large-scale, frequent, indiscriminate, and sometimes excessive application of disinfectants amid COVID-19 could pose a serious impact on the urban environment, biodiversity, and public health.^10^ The frequency (69.3%) and amount (74.2%) of cleaning product usage increased significantly since the pandemic, according to a survey in autumn 2020 in Turkey.^13^ Data according to the Centers for Disease Control and Prevention (CDC) of the United States show 20.4% more reports of human poisoning due to exposure to cleaning products and disinfectants following unsafe use in the first three months of COVID-19 outbreak in January 2020.^14^ While physical methods such as UV irradiation^15^ and heat treatment^7^ are effective in disinfecting surfaces, they are less practical for use on a daily basis and in public space.

Surface coatings incorporating certain metal elements, notably copper and silver, exhibit virucidal properties by actively breaking the disulfide bonds of virus proteins^16^ and/or releasing reactive oxygen species (ROS), which disrupt the structural integrity of virus.^4,17−19^ However, it is not always practical to apply such coating readily onto existing high-traffic objects such as a door handle due to the constraints of the manufacturing process (e.g., high temperatures and chemicals involved). Hygiene technologies and/or products that can effectively, continuously, yet sustainably function to minimize surface transmission of pathogens, with little impact on the environment, remain an unmet characteristic of most of the current designs and products, although sustainable cleaning products could be 110 billion U.S. dollars per annum in 2025.^20^

It has become clear that the respiratory liquid serves as the protective media and vehicle during the transmission of virus,^21^ which underpins the surface viability of respiratory virus such as SARS-CoV-2 in moist environments.^22,23^ A previous study shows a clear correlation between the evaporation kinetics of respiratory droplets on a surface and the virus persistence time.^24^ The fundamental principle of a different strategy for virus inactivation is thus to target the respiratory droplets as opposed to the virions within. The porous nature of a surface coating can facilitate an imbibition process that is much faster than the diffusion-limited evaporation, which spreads and drains the virus-containing droplets quickly.^24−26^ The virus is subsequently dried within the porous matrix^27^ and loses infectivity by up to 100-fold during drying.^28^ With the virus being trapped and adhering to the pores below the top surface, microbial transfer through finger touching or rubbing can also be significantly reduced.^4^ Such design principles for antimicrobial coatings, without the involvement of any chemical additives, are attractive as a passive surface hygiene approach to inhibit the transmission of infectious diseases. The limiting factor, nevertheless, is the limited scope of implementing such coating on an object currently in use in a sustainable and cost-effective manner. The antimicrobial efficiency of such a porous film as a function of exposure to materials in contact in real-life scenarios, e.g., food stain and cleaning agents, is an unknown factor for the permanent coating.

Inspired by clinical evidence and previous work on porous surface coating, we have developed a suite of cellulose-based surface films that are hydrophilic and porous, which could capture respiratory droplets immediately. The evaporation kinetics of water and a model respiratory fluid on the cellulose-coated surfaces were investigated. The efficiency of the porous films in reducing droplet contact transfer and the mechanical stability of the films were evaluated. The antiviral efficiency was tested by deploying an infectious SARS-CoV-2 culture. The film developed could serve as a submicrometer porous coating that offers multiple functions, e.g., antiviral, antimicrobial, and active carriers, so that the film could be used for high-traffic objects as an effective intervention for surface contact transmission.

## Results and Discussion

### Morphological Characteristics of a Microfibrillated Cellulose Film

**Figure 1:**
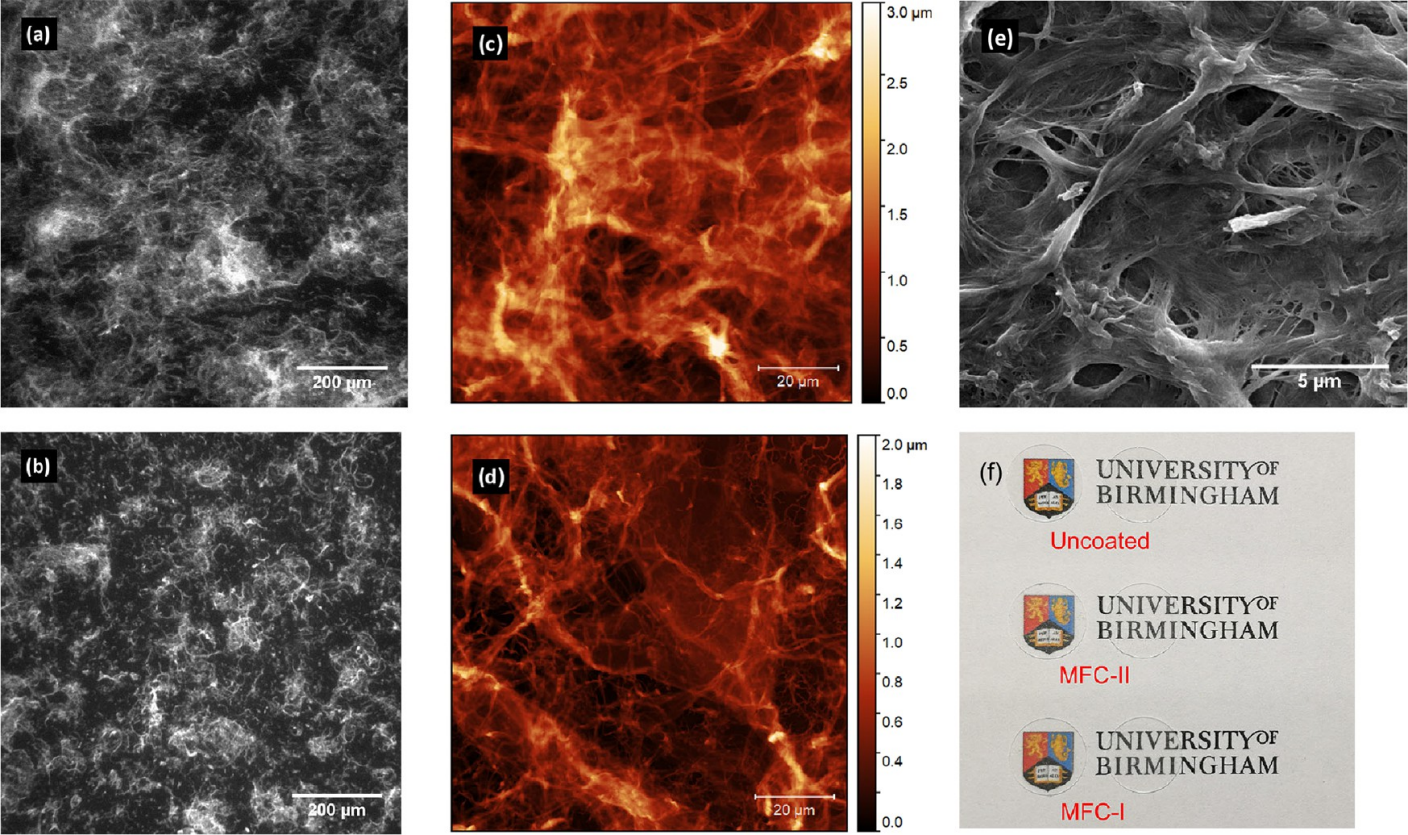
Microscopic characterization. Morphology of the cellulose thin films observed by an (a,b) optical microscope and (c,d) AFM. The sample in (a) and (c) was prepared by spin coating (i.e., MFC-I); the sample in (b) and (d) was by spraying (MFC-II). (e) SEM image showing a representative view of the porosity (scale bar: 5 μm). (f) Picture of uncoated glass discs and those coated with the cellulose thin films, demonstrating their optical transparency under natural light.

Porous films were fabricated on glass substrates by either spin coating or spraying an aqueous suspension of microfibrillated cellulose (MFC). A dry film was formed on the substrate within 10–30 s after applying the suspension. It is suggested that hydrogen bonds naturally form between the fibrils, which immobilize the network and enable sufficient adhesion to the substrate to ensure film durability. Figure 1 presents the morphology of two representative thin cellulose films with fine details. We observed a porous and interconnected weblike structure, consisting of individual cellulose fibrils and a small fraction of pulp bundles. The two coating methods produced coverage of 91% (spin coating, referred to as MFC-I) and 44% (surface spraying, MFC-II) on the substrate.

Based on the images acquired by atomic force microscopy (AFM), the prepared MFC-I and MFC-II films were 1.2 μm- and 300 nm-thick, respectively, rendering them barely noticeable to human eyes (Figure 1f). Surface and porosity parameters are summarized in Table 1. MFC-I possesses greater surface roughness but a lower mean pore size compared with MFC-II. MFC-I was prepared by spin coating whereby the MFC suspension was added dropwise onto a spinning substrate (6000 rpm). Due to the dynamic process of spin coating, we found that it was easier to form clusters of cellulose fibers on MFC-I than on MFC-II, leading to more surface irregularities and thus a greater roughness value. Nonetheless, the average pore size decreases as MFC fibers accumulate and network on the surface. It is worth noting that the morphological characteristics of the cellulose films in this work could be effectively modulated by varying the fabrication conditions. For example, an increased speed of the spin coating process reduced the roughness and increased the porosity of the resulting film, while an increasing amount of the initial cellulose suspension led to an increased surface roughness and reduced porosity levels (Figure S1, Supporting Information). It is also worth noting that the selection of thin-film specifications in this work was made with a primary consideration of demonstrating two possible coating fabrication methods rather than differentiating the antimicrobial efficiencies of thin films made with varied coating configurations. The spin coater allowed fine control of the coating process and the surface parameters of the resulting thin film, which would potentially have consequences for antimicrobial efficiency. However, spray coating would warrant practical feasibility for household use, as only an ordinary spray bottle filled with a MFC suspension is required. The resulting coating remains considerably tunable by adjusting operation parameters such as the MFC concentration, nozzle diameter, number of spray cycles, etc.

**Table 1:** Surface Parameters of the MFC Thin Films

	MFC-I	MFC-II
roughness *R*_a_ (nm)	247 ± 54	97 ± 10
waviness *W*_a_ (nm)	460 ± 135	144 ± 20
surface coverage	91%	44%
thickness (nm)	1200	300
porosity (% of the projected area)	32 ± 7	58 ± 9
mean pore size (μm)	5.8 ± 0.5	10.1 ± 2.3

### Surface Spreading and Evaporation of Liquid Droplets

Surfaces bearing virus-laden droplets can serve as media for the transmission of pathogens. Previous studies suggest that porous surfaces provide advantages in disrupting the surface viability of virus due to a much faster droplet evaporation, underpinned by a capillary imbibition effect, as opposed to the slow diffusion-limited evaporation on impermeable surfaces.^24^ As such, fabrication of porous and fluid-absorbing coatings could be an innovative strategy for antiviral surfaces and devices. They offer unique practical and environmental benefits missing in other antiviral solutions, such as metallic, polymer, and multilayered nanoparticle coatings.^29^

**Figure 2:**
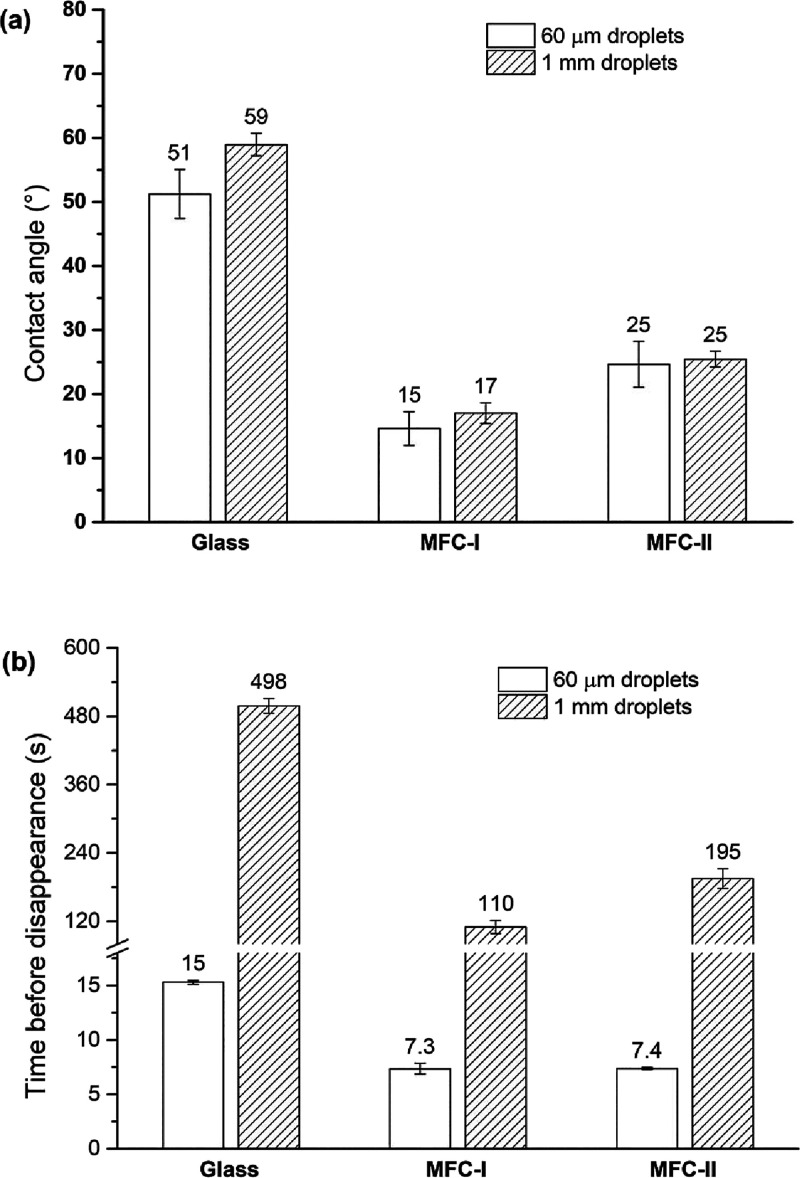
Contact angle measurements. (a) Initial contact angles of water droplets of two different sizes on planar glass and the MFC thin films. (b) Time before droplets disappeared from the surface, determined as the point when the droplet was no longer discernible by the contact angle goniometer.

Figure 2a compares the initial water contact angles of two distinct droplet sizes (60 μm and 1 mm in diameter) on the two cellulose thin films, with a clean and planar glass substrate as a control. Droplets of both sizes exhibited similar initial contact angles on each type of cellulose coating, evidencing that the droplet volume does not influence the interfacial energy. However, the two cellulose thin films prepared, MFC-I and MFC-II, showed much smaller contact angles, about 1/3 and 1/2 of that on the uncoated glass substrate, respectively. Apart from the difference in material composition, this reduction in contact angles could be attributed to the hydrophilic and thus more wettable surface of the cellulose architecture, which is consistent with the mechanism suggested by previous studies that the surface porosity could introduce an imbibition effect to distribute the water through the pores due to capillary force, facilitating evaporation at an increased rate.^24,30^ According to the model suggested by Starov and colleagues in describing capillary force-assisted spreading,^31^ the spreading time can be calculated with the following equation:
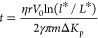
1where η is viscosity of the liquid, γ is liquid surface tension, *V*_0_ is the initial drop volume, l* is the maximum radius of the wetted region, L* is the maximum radius of the drop base, *r* is the scale of pore radii inside the porous layer, *m* is porosity, Δ is thickness of the porous layer, and *K*_p_ is permeability of the porous layer. The capillary pressure inside the porous structure, *p*_c_, can be estimated as
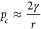
2

It suggests that small pore radii would result in great capillary pressure within the porous layer and in turn effectively shortens the spreading time of a liquid drop of a given volume.

Rapid droplet spreading and imbibition on the cellulose thin films were observed *in situ* using a contact angle goniometer (Figure 2b). A droplet of 60 μm diameter remained discernible for 15 s on a planar glass surface that allows solely evaporation, of which the duration was halved on both cellulose films (MFC-I and MFC-II). The effect of surface porosity on the water droplet of 1 mm diameter is much more significant, and the duration before disappearance was reduced from 498 s on a planar glass to 110 s when cellulose films were in place, which is because water was wicked into the porous surface of the cellulose films in the latter case. The results in Figure 2b suggest that the cellulose thin films can effectively accelerate the loss of water by wicking the aqueous droplets; the significance of such reduction is to minimize the time window for transfer of pathogens upon contact to take place.

**Figure 3:**
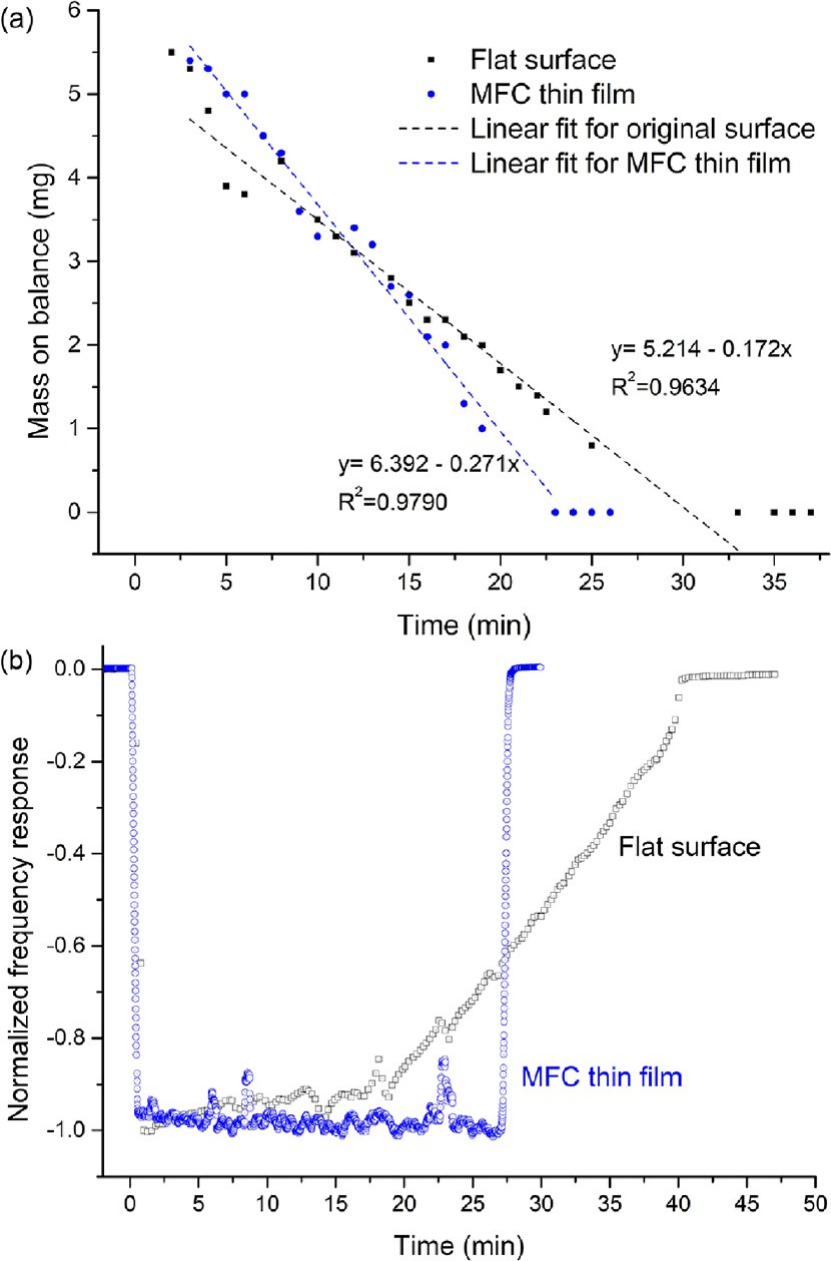
*In situ* evaporation kinetics measured by (a) gravimetry and (b) QCM. Water droplets of 5 μL were placed on a mass balance and a quartz crystal surface and left evaporating until full dryness was achieved.

The effect of the porous cellulose film on water evaporation kinetics was measured *in situ* using a laboratory balance with a 0.1 mg sensitivity. All experiments were conducted under ambient temperature (∼21 °C) and 48–50% relative humidity. Figure 3a presents the gravimetric reading of a water droplet (initial volume of 5 μL) as a function of time: the water drop evaporated clearly faster (0.271 vs 0.172 mg·min^–1^) on the cellulose-coated surface than on a planar, uncoated glass substrate. The linearity of the mass loss on the planar but solid substrate (black squares and fitted line) indicates an evaporation process governed by diffusion, with the highest evaporation flux occurring at the contact line.^24,32^ The evaporation rate of sessile droplets shall thus be proportional to the contact radius.^33^ The changed evaporation kinetics observed on the cellulose film (blue dots and fitted line) reflects the accelerated evaporation discussed earlier.

The evaporation process of the sessile droplets was measured using a quartz crystal microbalance (Figure 3b) that is highly sensitive to changes in the temporal droplet radius.^34,35^ We observed that the droplet evaporating on the planar surface (without cellulose coating) initially followed a constant contact radius (CCR) regime, during which the liquid–solid contact line was pinned while the contact angle and the droplet height decreased as the evaporation continued followed by a depinning period where the contact line contracted until the evaporation was completed. In contrast, for the substrate covered by the cellulose thin film, the droplet remained evaporating in a constant contact radius mode for most of its lifetime due to the capillary-driven spreading and imbibition in the presence of the porous medium, leading to a much accelerated evaporation process.^32^ The difference in evaporation time and associated kinetics between the two MFC film samples was not noticeable and hence not included.

### Antimicrobial Testing

**Figure 4:**
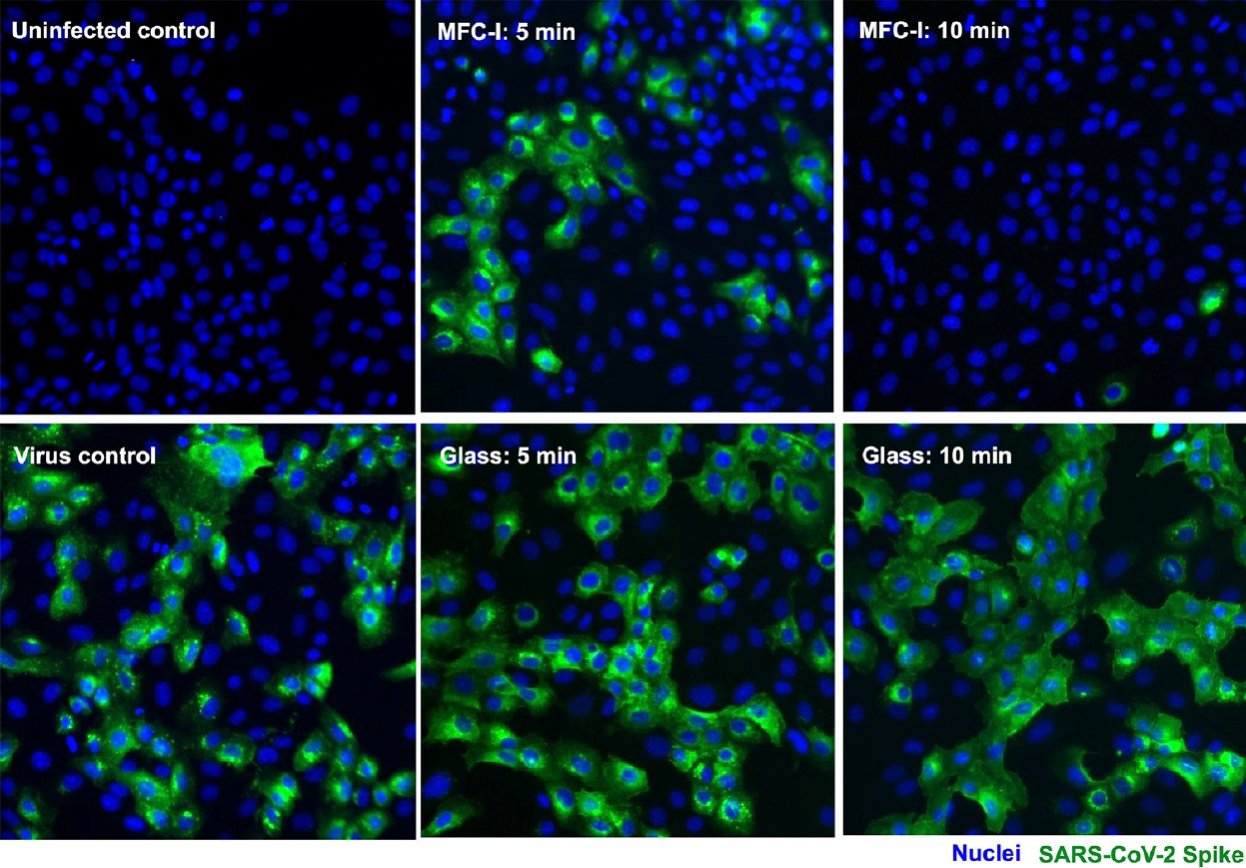
Cell line images showing the *in vitro* testing of SARS-CoV-2 on the cellulose thin film and the glass surface. Droplets of virus-containing culture medium were left on the porous cellulose film (MFC-I) and the planar glass substrate. Any infectious particles were recovered from these surfaces either 5 or 10 min after droplet deposition and transferred to target cells for infection. Hoechst was used to visualize the nucleus of Vero cells (blue). Infected cells are indicated by SARS-CoV-2 spike protein (green).

**Figure 5:**
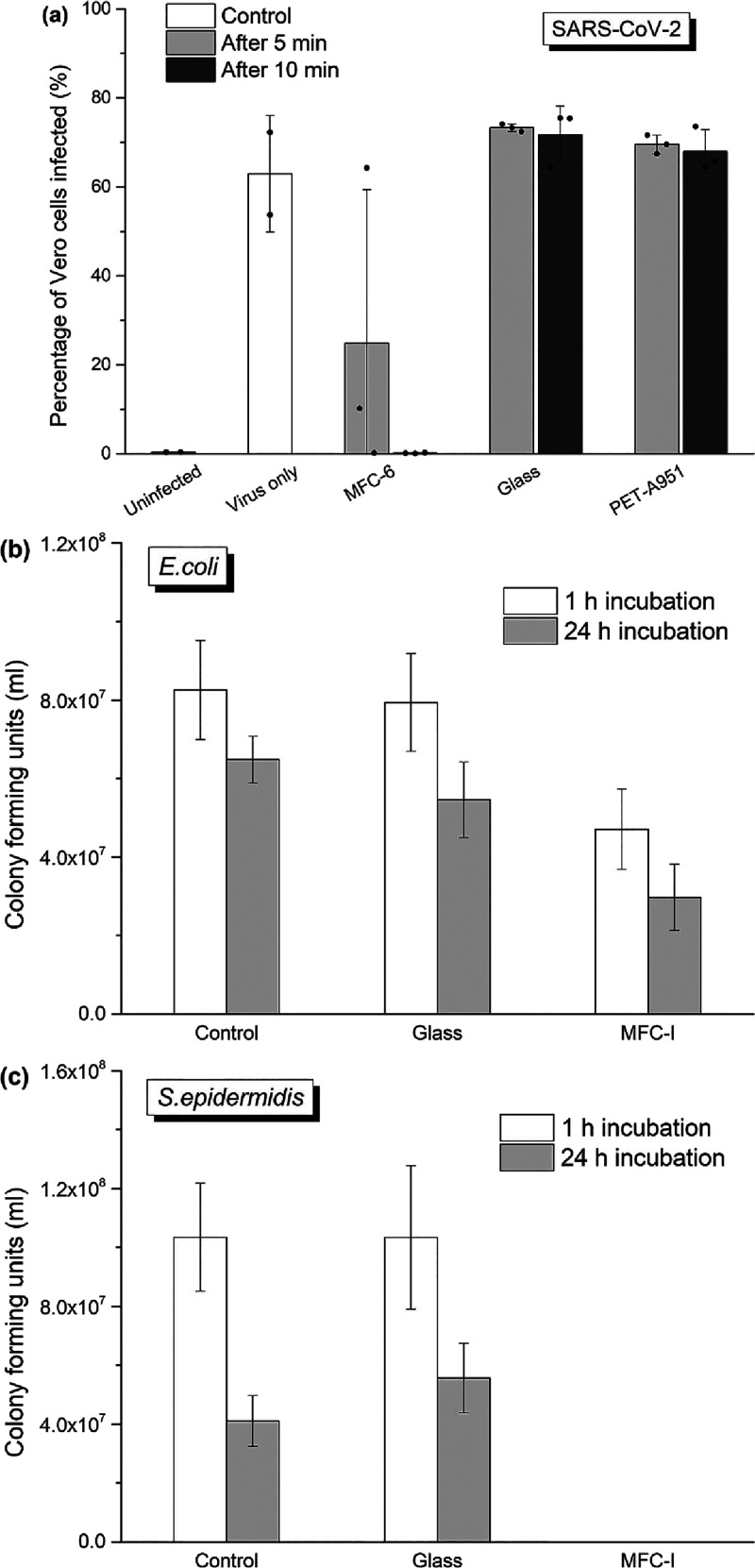
Antimicrobial testing results. (a) *In vitro* inactivation testing of SARS-CoV-2 on the cellulose film and the glass substrate. Droplets of virus-containing culture medium were deposited on the porous cellulose film (MFC-I) and planar laboratory-grade glass coverslips. Any infectious particles were recovered from these surfaces either 5 or 10 min after droplet deposition and transferred to target cells for infection. A control group (i.e., virus medium added to the cell culture directly without pre-exposure on any substrates) is also shown. (b,c) Viability of two representative types of microbes (*Escherichia coli* (*E. coli*) and *Staphylococcus epidermidis* (*S. epidermidis*)) after incubation on the cellulose thin film and glass for 1 and 24 h.

The ability of the cellulose coating to inhibit surface transmission of SARS-CoV-2 was studied through *in vitro* infection of Vero cells as demonstrated in a previous study.^36^ To replicate the surface transmission process of fomites via contact, 1 mm-diameter droplets of a virion-containing culture supernatant were deposited on the testing substrate and left in ambient conditions for 5 or 10 min before virus recovery was carried out, followed by cell infection procedures to determine the amount of virions (infectious particles) recovered from the surface. Figure 4 shows cell line images after the *in vitro* testing where the quick inactivation of SARS-CoV-2 in the presence of the cellulose thin film can be clearly seen. Figure 5a shows the infectivity of SARS-CoV-2 as a function of substrate characteristics and droplet residence time. Direct contact of the droplets with Vero cells (“virus only”) led to an infection rate of approximately 70%. However, a three-fold reduction of infectivity was observed when the droplets were left on the cellulose thin film (MFC-I) for 5 min. After a residence time of 10 min on the cellulose thin film, the infectivity was statistically nil and comparable to the uninfected group, which confirms a major reduction of surface transmission of virus. In contrast, the infectivity was comparable to the control group if the virions were recovered from a planar and solid glass substrate, regardless of the residence times of 5 or 10 min.

The effectiveness of the cellulose film in inactivating bacteria was also assessed using two representative pathogens: *E. coli* (Figure 5b) and *S. epidermidis* (Figure 5c), alongside two benchmarks: control (bacteria incubated in a broth) and those incubated on glass substrates. While the bare glass surface showed statistically no effect on the viability of both bacteria incubated on its top when compared to the control group, the viability of *E. coli* was reduced by 43 and 54% when incubated on the cellulose thin film for 1 and 24 h, respectively. Meanwhile, we found that *S. epidermidis* was especially vulnerable to the cellulose-coated surface, with a complete loss of viability after incubation for 1 h.

### Contact Transfer and Mechanical Stability

**Figure 6:**
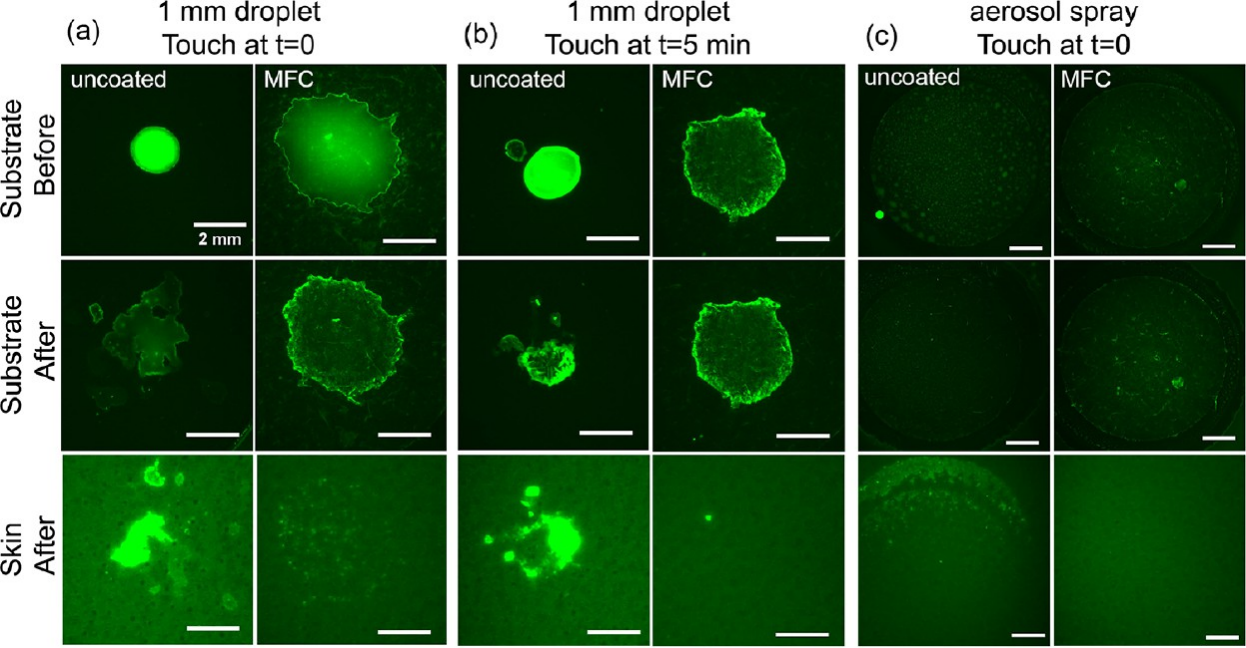
Contact transfer tests of artificial saliva. (a,b) Artificial saliva droplets and (c) aerosol were deposited on glass substrates with or without cellulose films (MFC) followed by contact on the normal direction with an artificial skin for a set time. (a) Contact was made immediately after the deposition of a 1 mm droplet. (b) Contact was made 5 min after the deposition of a 1 mm droplet. (c) Aerosol of the artificial saliva was sprayed onto the substrates, and contact was made immediately following the aerosol spray. Scale bars of 2 mm in all images.

A recent study reported that SARS-CoV-2 can be transferred to an artificial finger through a brief and light contact with contaminated surfaces, and the transfer of virus was found to be much less from porous than from nonporous substrates. The authors postulated that porosity plays the key role by allowing penetration and thus less transfer of respiratory liquid, which in turn reduces the transfer of virus.^4^ In the present study, we performed a series of contact measurements between an artificial skin and glass substrates with or without the cellulose films, in the presence of respiratory liquid (artificial saliva), of which fluorescence micrographs are presented in Figure 6.

Figure 6a shows that upon deposition (top line in the plot) of a 1 mm-diameter artificial saliva droplet on the host substrates, the droplet spread 400% wider on the cellulose-coated surface compared with that on the uncoated glass substrate. Contact was then made immediately following the droplet deposition. After contact (middle and bottom rows in Figure 6a), an intense and large area of fluorescence was observed on the artificial skin, which must have come from the uncoated substrate, suggesting a considerable amount of liquid was transferred upon contact. In contrast, the artificial skin showed only little fluorescence signals after contacting the cellulose-coated surface, of which the intensity is significantly weaker, and the contaminated area is approximately 90% less compared to that out of the flat glass surface.

Figure 6b shows the scenario where a droplet residence time of 5 min was allowed before surface contact was made. Prior to the contact, the droplet on the glass substrate was still hydrated, while the droplet on the cellulose-coated substrate was fully dry already, based on their morphologies shown in the micrographs. Consequently, the artificial skin in contact with uncoated glass was once again heavily soiled. In contrast, we observed nearly zero fluorescence signals on the artificial skin that was in contact with the cellulose film, demonstrating the excellent ability to “lock” liquid droplets and mitigate potential transfer upon contact. Figure 6c shows the contact transfer performance of the same substrates that were subject to sprays of artificial saliva aerosol. Figure 6c and the quantitative analysis (Figure S2) suggest that the cellulose thin film is equally effective in suppressing the contact transfer of respiratory aerosols.

**Figure 7:**
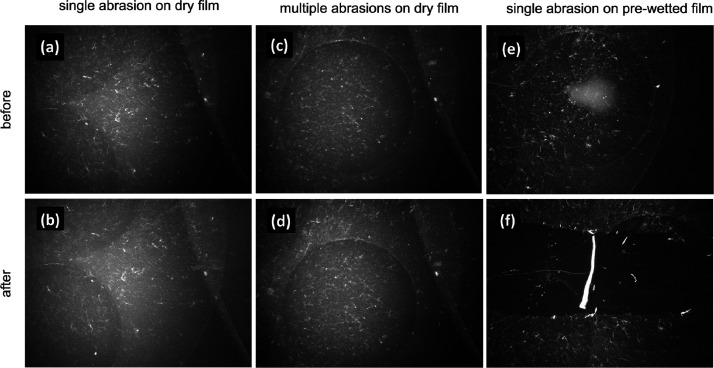
Mechanical stability tests. Morphology of the cellulose thin films before and after scraping tests under different wear conditions. (a,b) Single pass; (c,d) reciprocating, multiple passes; (e,f) single pass, against a prewetted cellulose film. The tests were performed using an artificial skin covering the whole substrate surface.

The mechanical stability of the cellulose thin films was evaluated by means of scraping tests against an artificial skin under a normal compression load (loading history is shown in Figure S4). Images of the exact locations prior to and after abrasion tests were acquired by a bright-field optical microscope. As shown in Figure 7a–d, the cellulose thin films did not show any noticeable disruption after the abrasion under dry conditions, even after multiple cycles of back and forth scraping. The satisfactory mechanical strength of the thin film is attributed to the hydrogen bonding between cellulose fibrils and the supporting substrate (glass in the present work), which sufficiently immobilizes the network of cellulose fibrils, providing a considerable resistance to occasional abrasions while it is dry. This suggests the suitability of such coating for high-traffic objects such as door handles and handrails that are regularly in contact with the human skin. The cellulose thin film, however, can be easily removed with a single abrasion, with the presence of a small quantity (1 mm droplet in the present work) of water (Figure 7e,f). The results suggest that the cellulose film can not only be easily applied on common surfaces and maintain its strength and functionality in dry conditions but also exhibit great removability once wetted, making it convenient and suitable for daily cleaning and disinfection practice.

### Mechanisms of Action

**Figure 8:**
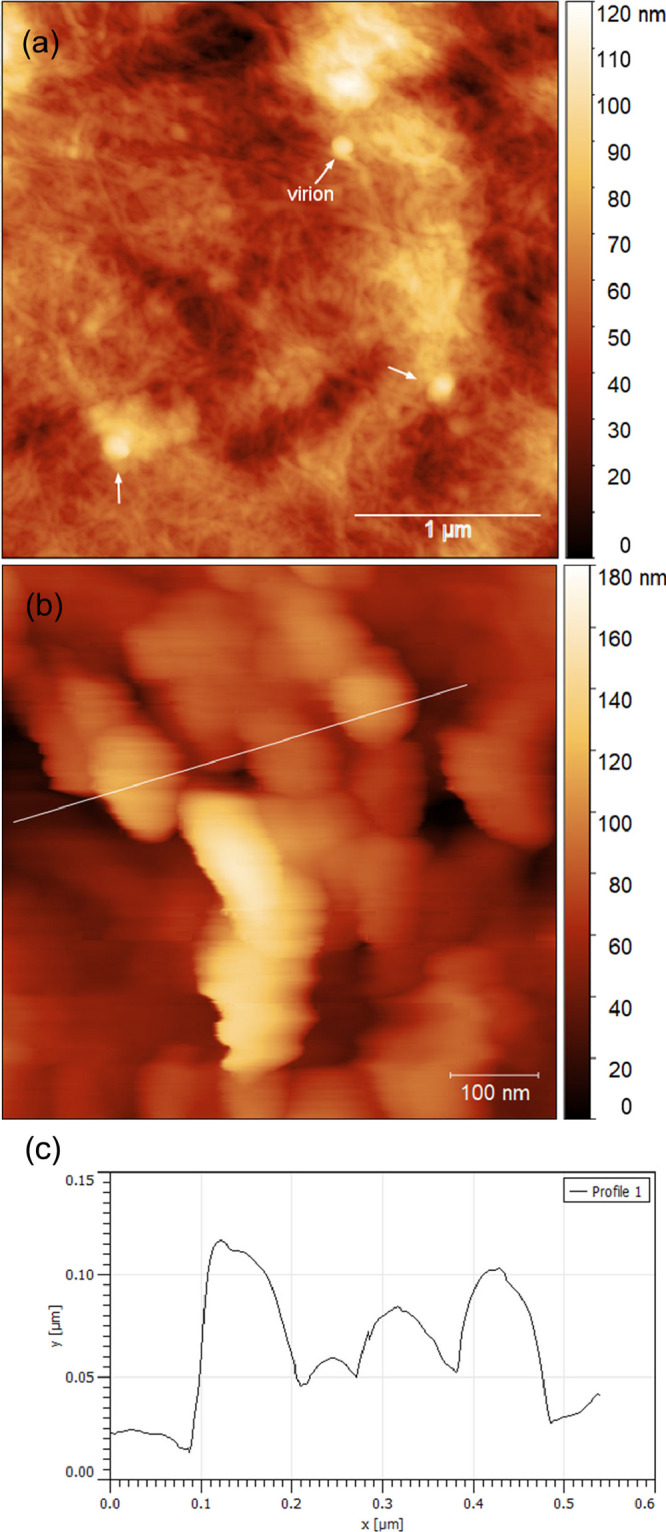
AFM images of (a) 3 × 3 μm scan size and (b) 500 × 500 nm scan size; (c) corresponding sectional analysis of SARS-CoV-2 viral particles present on the cellulose-coated substrate.

**Figure 9:**
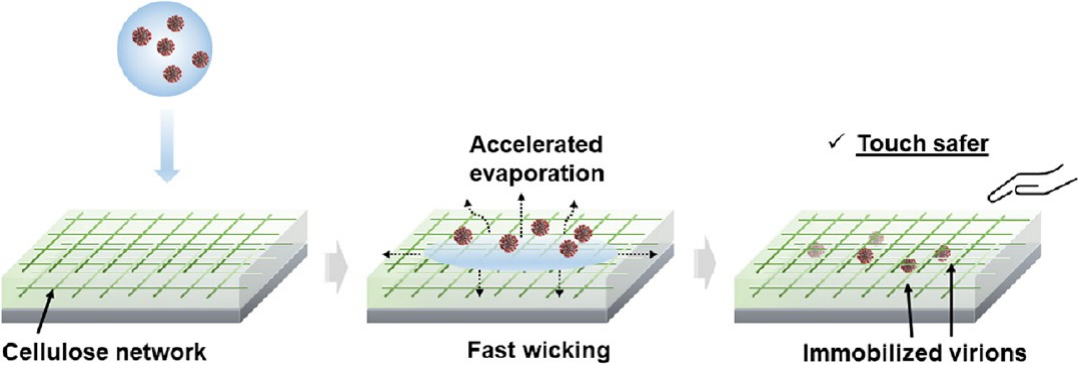
Schematic diagrams illustrating the proposed mechanism of action against respiratory droplets containing virions.

Figure 8a shows the cellulose thin film bearing dried SARS-CoV-2-containing medium, acquired by AFM; some spherical particles were found dispersed within the network of cellulose fibrils. A high-resolution scan of the particles (Figure 8b,c) revealed dimensions matching those of SARS-CoV-2. The AFM results support our antiviral data that a rapid and effective virus inactivation action was introduced to the cellulose film. Figure 9 illustrates the possible antiviral mechanism of the cellulose thin film in this work: the fast virus inactivation effect of the MFC porous film can be attributed to the accelerated drop spreading and evaporation, as demonstrated in Figures 2 and 3. It has been well-documented that the persistence of SARS-CoV-2, among other respiratory coronaviruses, is positively correlated with the surrounding water and moisture conditions.^22,23^ Several studies reported that liquid droplets can spread quickly and evaporate in seconds on porous and hydrophilic surfaces, as opposed to the long evaporation time (minutes) on impermeable solid surface because of the imbibition effect induced by the surface porosity.^24,26^ In the present work, the virus-containing droplets were found to dry completely after 5 min on the cellulose-coated substrate, leaving the virions exposed to the ambient environment and prone to disruption. As a contrast, liquid droplets of similar initial size remained partially hydrated on a nonporous glass substrate, even after 10 min. More importantly, upon wicking of the deposited respiratory liquid, the highly porous cellulose network can act as an effective “trap”, which immobilizes the virions and prevents them from being picked up from the contaminated surface by further vortex virus recovery or hand touching as already demonstrated in the preceding sections. Figure 9 presents a set of schematic diagrams to illustrate the possible mechanisms. This entrapment mechanism is in good agreement with a recent study whereby nanocellulose hydrogels and self-standing films were shown effective in capturing nano- (100 nm) and microplastics (≥1 μm) from aqueous environments, owing to the synergistic feature provided by the highly hygroscopic and high-surface-area nanocellulose network.^37^

The antibacterial effect of the cellulose film is also considered to have benefited from its hydrophilic and porous nature. Although the increased surface roughness resulted from the porous coating would likely facilitate bacterial adherence to the cellulose film, the imbibition effect induced by the capillary force, as explained by eqs 1 and 2, has the ability to remove any liquid medium surrounding bacteria. It is very likely that the withdrawal of the liquid, evidenced by our evaporation study, causes an imbalanced osmotic pressure at the bacterial membrane, which is effectively a hypertonic condition with a low water concentration in close proximity to the microbes. The antimicrobial effect of porous material had been reported in the past,^38,39^ e.g., Aviat and colleagues carried out a comprehensive review of microbial safety of wood in contact with food^39^ and came to the conclusion that the surface cavities of wood could trap microorganisms and generate unfavorable conditions for their survival, which is consistent with the finding in the present work. In another review article by Munir and coworkers, the antimicrobial characteristics of wood materials were confirmed.^38^ The authors reviewed different methods available to differentiate the possible nature of the antimicrobial properties of wood materials: porous structures or actions of molecules extracted from wood on bacteria and fungi. Although there was no conclusive suggestion on the best methods to be used, the antimicrobial effect of porous wood was the basis, which again supports our finding in the present work. The difference between the viability of *E. coli* and *S. epidermidis* could be attributed to the variation in the structural characteristics between Gram-positive and Gram-negative bacteria.

Lastly, we would like to highlight that the results of our present work, alongside a number of recent studies^25,26,40,41^ by Ducker and coworkers amid COVID-19, indicate explicitly that hydrophilic and porous surface coatings could be an effective strategy against the surface transmission of respiratory pathogens. Unlike antimicrobial surface coating approaches whereby nanoparticles are incorporated in a matrix, the cellulose thin film reported in this study provides a practical and sustainable antimicrobial solution. The high surface area and abundant hydroxyl sites naturally present on the cellulose fibrils can potentially facilitate enhanced antimicrobial effects through additional surface functionalization such as charged polymers or natural enzyme compounds.

## Conclusions

A sustainable and effective antimicrobial surface thin film based on microfibrillated cellulose has been developed in the present work. The porous cellulose film, with submicrometer thickness, is barely noticeable to human eyes but can effectively render the surface hydrophilic (a three-fold reduction in water contact angles) and accelerate (more than 50% faster) the evaporation of respiratory droplets, which is shown by QCM analysis. The film exhibits not only a rapid inactivation effect against SARS-CoV-2 in 5 min following the deposition of the virus-containing droplets but also an exceptional ability to reduce contact transfer of liquid, e.g., respiratory droplets, onto surfaces such as an artificial skin by more than 90% as shown by contact transfer tests. Additionally, the thin film is also effective in inhibiting the growth of both Gram-negative and Gram-positive bacteria (*E. coli* and *S. epidermidis*). Moreover, with strong attachment to the substrate, the cellulose film can provide nearly 100% resistance to skin scraping in dry conditions while good removability once wetted, suggesting its practical suitability for daily use. Importantly, the thin film can be formed on solid substrates readily by spraying and requires solely a simple formulation of a plant-based cellulose material. The flexibilities in controlling the formulation, cellulose surface chemistry, and coating process offer a powerful platform to deliver appropriate functionalities. These notable advantages of the cellulose film offer a scalable, affordable, and green solution to mitigate the transmission of infectious diseases that spread via the respiratory fluid.

## Methods

### Materials

A microfibrillated cellulose (MFC) aqueous slurry (solid content of 7 wt %, FiberLean Technologies Ltd., UK) was diluted with ethanol (v/v = 1:5) and homogenized (SHM1 homogenizer, Stuart, UK) for 3 min before use. Polyethyleneimine (PEI; 181978), phosphate-buffered saline (PBS; P4417), mucin (type I-S; M3895), bovine serum albumin (BSA; A9647), and tryptone (T9410) were purchased from Sigma-Aldrich. Fluorescence dye (Alexa Fluor 488 C5 maleimide) was purchased from Thermo Fisher Scientific.

An artificial saliva solution was prepared in compliance with the international standard ASTM E2197 and formed with three types of proteins: (i) high-molecular-weight proteins (e.g., bovine serum albumin, BSA), (ii) low-molecular-weight peptides (e.g., tryptone), and (iii) mucous material (e.g., mucin). In a typical process, the three protein solutions were prepared separately by adding 0.5 g of BSA, 0.5 g of tryptone, and 0.04 g of mucin into 10 mL of PBS individually. Each of them was passed through a 0.22 μm pore diameter membrane filter, aliquoted, and stored at either 4 ± 2 or −20 ± 2 °C. To obtain 500 μL of artificial saliva solution, we added 25 μL of BSA, 100 μL of mucin, and 35 μL of tryptone to 340 μL PBS and mixed them well (magnetic stirring, 30 min) before use. The concentration of mucin in the resulting artificial saliva was 0.8 mg·mL^–1^.

Nutrient agar (OXOID Ltd.) contained (per liter of deionized water) 1 g of “Lab-Lemco” powder, 2 g of yeast extract, 5 g of peptone, 5 g of sodium chloride, 15 g of agar (pH 7.4). Luria–Bertani broth contained (per liter of deionized water) 10 g of sodium chloride, 10 g of tryptone, and 5 g of yeast extract. Phosphate-buffered saline (OXOID Ltd.) contained (per liter of deionized water) 8 g of sodium chloride, 0.2 g of potassium chloride, 1.2 g of disodium hydrogen phosphate, and 0.2 g of potassium dihydrogen phosphate. All solutions were autoclaved for 20 min before use.

### Thin-Film Fabrication

Glass coverslips (φ of 10 mm, thickness of 0.16–0.19 mm, Fisher Scientific, UK) were cleaned with ethanol and then placed in an oxygen plasma chamber (HPT-100, Henniker Plasma) at an oxygen flow rate of 10 sccm for 5 min. Polyethyleneimine solution (1% w/v in H_2_O) of 70 μL was placed on the cleaned substrate. The loaded substrate was spun at 600 rpm for 30 s on a spin coater (SPIN150i, APT GmbH), then accelerated at 500 rpm/s to 4000 rpm, and spun for 60 s. MFC thin films were fabricated on such pretreated surfaces by two different approaches, namely, spin coating and spray coating. In the case of spin coating, 400 μL of an MFC suspension was added dropwise onto the sample spinning at 6000 rpm. In the case of spray coating, a manual cosmetic atomizer was used to apply the MFC suspension onto the stationary sample. A total of 40 sprays were made to obtain uniform coverage.

### Surface Characterization

The surface morphology of the thin films was examined by an atomic force microscope (AFM, Multimode, Bruker) with a tapping mode cantilever (NCHR-20, Apex Probes Ltd.) and a scanning electron microscope (Philips XL-30 FEG ESEM). Surface parameters were extracted from the scans (over a range of 1.4 × 1.0 mm) by a white-light interferometer (WLI). Porosity levels of the thin films were evaluated semiquantitatively using the image processing program Gwyddion and the integrated Watershed algorithm.^42^ Contact angle measurements were carried out using two distinct droplet sizes, i.e., 60 μm and 1 mm in diameter (100 pL and 0.5 μL in volume), for which an optical contact angle instrument equipped with a picoliter dosing system (PDDS, DataPhysics Instruments GmbH) and a generic contact angle goniometer (Ossila Ltd.) were employed, respectively.

### Quartz Crystal Microbalance

The evaporation behavior of water droplets was studied using silicon dioxide-coated QCM sensors (5 MHz 14 mm Cr/Au/SiO_2_, Quartz Pro, Sweden). The surfaces of the sensors were pretreated, and MFC thin films were fabricated as mentioned in the “Thin-Film Fabrication” section. Water droplets of 5 μL were placed onto the QCM sensor by a micropipette. The frequency and energy dissipation history through the quartz sensors during the droplet landing and evaporation events was simultaneously monitored and recorded by a quartz crystal microbalance (NEXT, openQCM, Italy).

### Antiviral Analysis

We attempted to replicate virus droplets as in a sneeze from an infected person whereby 0.5 μL drops of medium containing SARS-CoV-2 (England 2 stock 10^6^ IU·mL^–1^ (kind gift from Christine Bruce, Public Health England)) were placed on the testing materials and left at room temperature for either 5 (semidry) or 10 min (dry). The deposited drops were evident immediately in the porous materials. We then retrieved any remaining infectious virus from the treated surfaces using 50 μL of cell culture medium on top of the viral drops, which were transferred to target cells for infection. We measured infection in Vero cells at 48 h, by scoring the percentage of spike-expressing cells.

The Vero cells were washed with PBS, dislodged with 0.25% trypsin–EDTA (Sigma Life Sciences), and seeded into 96-well imaging plates (Greiner) at a density of 10^4^ cells per well in culture media (Dulbecco’s modified Eagle medium (DMEM) containing 10% FBS, 1% penicillin and streptomycin, 1% l-glutamine, and 1% nonessential amino acids). Cells were incubated for 24 h to allow time for adherence and were fixed in ice-cold methanol after infection. Cells were then washed in PBS and stained with rabbit anti-SARS-CoV-2 spike protein, subunit 1 (The Native Antigen Company), followed by an Alexa Fluor 555-conjugated goat antirabbit IgG secondary antibody (Invitrogen, Thermo Fisher). Cell nuclei were visualized with Hoechst 33342 (Thermo Fisher). Cells were washed with PBS and then imaged and analyzed using a Thermo Scientific CellInsight CX5 high-content screening (HCS) platform. Infected cells were scored by perinuclear fluorescence above a set threshold determined by positive (untreated) and negative (uninfected) controls.

### Bacterial Testing

Strains of *E. coli* and *S. epidermidis* were incubated in 10 mL of L-B broth overnight in a 37 °C incubator with shaking at 150 rpm. Both species were pelleted and washed with 10 mL of PBS solution twice and suspended in PBS to an OD_600_ of 0.1 (*E. coli*: 8.5 × 10^7^ cells/mL, *S. epidermidis*: 10.3 × 10^7^ cells/mL). To the coated slides, 20 μL of the bacterial culture was added, and slides were placed in 24-well plates, which were sealed with a parafilm and incubated at 30 °C for either 1 or 24 h. After the set amount of time, surviving bacteria were recovered from the slides as follows. (i) Slides were placed into 0.7 mL of PBS in 15 mL falcon tubes and vortexed for 30 s. (ii) Slides were further physically scraped with a spatula, and the residue was mixed into the respective 0.7 mL solution from (i). (iii) Samples were then sonicated for 3 × 1 min in a bath sonicator (GT Sonic, 40 Hz, 100 W). Serial dilutions were then performed, and 10 μL of the final dilution was pipetted onto nutrient agar plates, which were left to soak into the agar for 30 min. Plates were then incubated at 37 °C overnight, after which colonies were counted to determine the antibacterial effect of the coatings.

### Contact Transfer and Durability Testing

To evaluate the transfer of respiratory liquid from the substrate, a piece of an artificial skin was pressed against the sample surface either immediately following the loading of artificial saliva droplets or after a residence time of 5 min. Both 1 mm-diameter droplets and aerosols of artificial saliva were employed. For the case of aerosol, a nebulizer (Omron C28P) ejected aerosolized droplets (mass median aerodynamic diameter of 3.0 μm) toward the sample for 30 s. The substrate, either uncoated or MFC-coated, was attached to a glass slide using carbon adhesive discs. The artificial skin was fixed on one end of an instrumented arm (Forceboard, Industrial Dynamics, Sweden AB), which in each test was driven smoothly toward the sample until a contact force of 2 N was reached. The artificial skin was then retracted from the sample surface. Each touch cycle lasted for approximately 5 s. Figure S1 shows the setup. The touched sample surface and the artificial skin counter surface were both examined under a fluorescence microscope (Leica Z16 APOA). The artificial saliva solution was stained by Alexa Fluor 488, which facilitates the observation and quantification of the mucin contaminations on each side. The areas of the fluorescent protein stains after the touch were measured via image processing using ImageJ.

Lateral scraping tests were performed to assess the mechanical stability of the thin films under both dry and wet conditions. A piece of the artificial skin was fixed on the level of the force board and lowered onto the substrate until normal force loads of 2 and 4 N were reached. Both one-pass and reciprocating multipass tests were performed in an ambient environment. An additional set of scraping tests were carried out on MFC thin films that were prewetted by placing a drop (0.5 μL, 1 mm diameter) of the artificial saliva. For all scraping tests, glass substrates of 25 mm diameter were used instead of the ones of 10 mm in the contact transfer tests.

## Supporting Information Available

Surface characteristics as a function of spin coating processing parameters; quantification of transferred mucin on the artificial skin from surfaces preloaded with model respiratory droplets; contact transfer setup; representative result of contact transfer (PDF)
