# Supplementary material for: Porous Cellulose Thin Films as Sustainable and Effective Antimicrobial Surface Coatings
Source: ACS Appl Mater Interfaces. 2023 Mar 29;15(17):20638–48. doi: 10.1021/acsami.2c23251 (PMC10165601; doi:10.1021/acsami.2c23251)
Supplement: Supplementary file 1 — am2c23251_si_001.pdf [file am2c23251_si_001.pdf]

Supplementary Material for

Porous cellulose thin films as sustainable and effective antimicrobial surface coatings

Shaojun Qi[1], Ioannis Kiratzis[1], Pavan Adoni[1], Aekkachai Tuekprakhon[2], Harriet James Hill[2], Zania Stamataki[2], Aneesa Nabi[1], David Waugh[3], Javier Rodriguez Rodriguez[1], Stuart Matthew Clarke[4], Peter J Fryer[1], and Zhenyu J Zhang[1]*

[1]School of Chemical Engineering, University of Birmingham, Birmingham B15 2TT, UK

[2]Institute for Immunology and Immunotherapy, University of Birmingham, Birmingham B15 2TT, UK

[3]School of Mechanical, Aerospace and Automotive Engineering, Coventry University, Coventry CV1 2JH, UK

[4]Yusuf Hamied Department of Chemistry, Cambridge University, Cambridge CB2 1EW, UK

* Corresponding author. E-mail Z.J.Zhang@bham.ac.uk

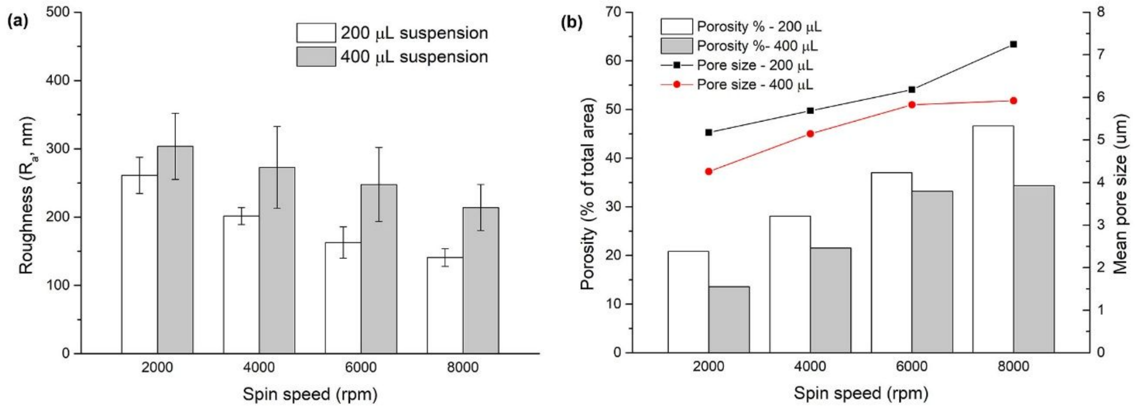

Figure S1. Tunable surface parameters of the resulting MFC thin films. a, Surface roughness (R_a) as a function of spinning speed (from 2000 to 8000 rpm) and amount of applied MFC suspension (200 or 400 μL) during a typical spin coating process. b, Effect of the spin coating conditions on the porosity level of the obtained MFC films.

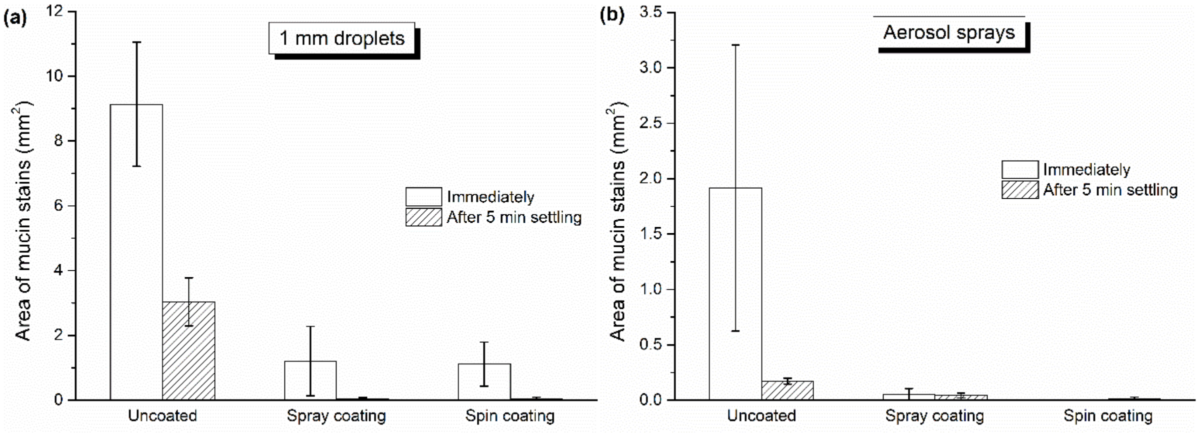

Figure S2. Quantification of transferred mucin on the artificial skin from the surfaces pre-loaded with (a) 1 mm droplets and (b) aerosol sprays.

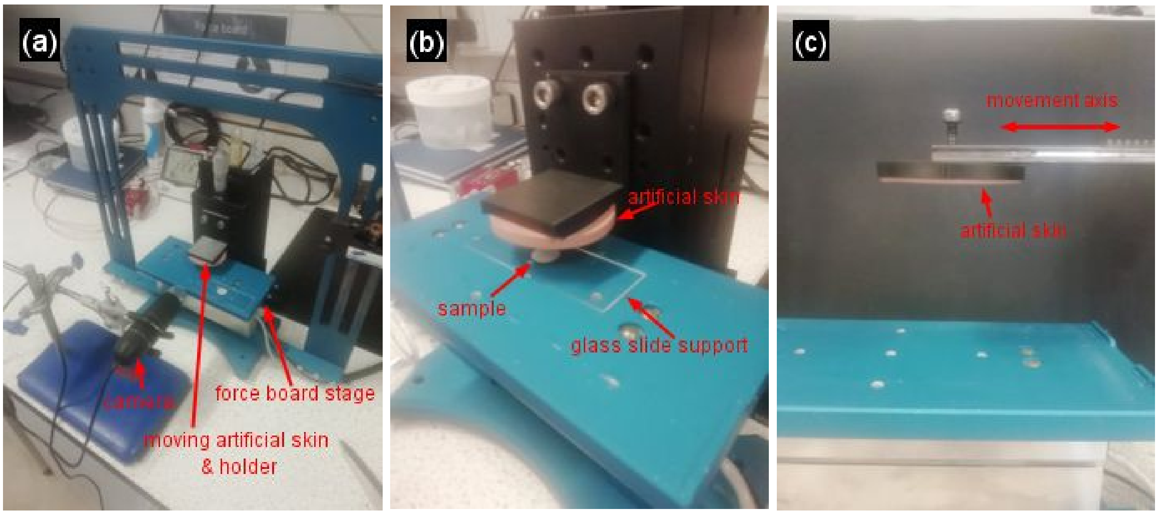

Figure S3. The contact transfer (a-b) and scraping (c) test set-up.

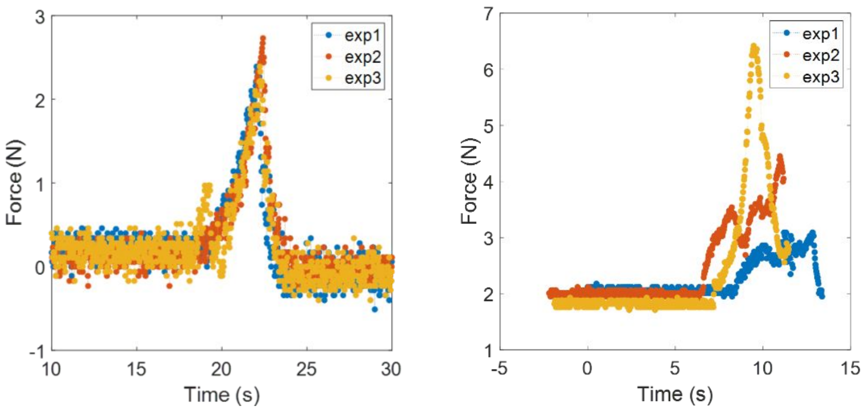

Figure S4. Representative result demonstrating the normal force history during (left) contact transfer and (right) scraping tests.
